# Substance P Increases the Excitability of Dorsal Motor Nucleus of the Vagus Nerve *via* Inhibition of Potassium Channels

**DOI:** 10.3389/fnins.2022.867831

**Published:** 2022-04-15

**Authors:** Eunhee Yang, Woojin Kim, Yong Seek Park, Young-Ho Jin

**Affiliations:** ^1^Department of Physiology, School of Medicine, Kyung Hee University, Seoul, South Korea; ^2^Department of Physiology, College of Korean Medicine, Kyung Hee University, Seoul, South Korea; ^3^Department of Microbiology, School of Medicine, Kyung Hee University, Seoul, South Korea

**Keywords:** chemotherapy, nausea and vomiting, neurokinin receptor, acid-sensitive potassium channel, leak potassium channel, substance P

## Abstract

Increases in the substance P (SP) concentration in the medial portion of the dorsal motor nucleus of the vagus nerve (mDMV) in the brainstem are closely associated with chemotherapy induced nausea and vomiting (CINV). However, the underlying cellular and molecular mechanisms of action are not well understood. In this study, we investigated the effects of SP on mDMV neurons using whole-cell patch-clamp recordings from rat brainstem slices. Application of different concentrations of SP induced tonic and phasic responses. Submicromolar concentrations of induced an inward shift of the holding current by increasing membrane input resistance. The response was mimicked by acidification of the extracellular solution and inhibited by a neurokinin type 1 receptor antagonist. These responses have equilibrium potentials close to the K^+^ equilibrium potential. In addition, a TWIK-related acid-sensitive K^+^ channel 3 (TASK-3) inhibitor, PK-THPP, induced responses similar to those produced by submicromolar SP concentrations. Micromolar concentrations of SP facilitated γ-aminobutyric acid (GABA) release but diminished glutamate release; these changes were blocked by a GABA_*B*_ receptor antagonist and a neurokinin type 3 receptor antagonist, respectively. In current-clamp recordings, submicromolar SP concentrations increased neuronal excitability by depolarizing membrane potentials. However, neither the increase in SP concentration to the micromolar range nor the addition of GABA_*A*_ and ionotropic glutamate receptor antagonists affected neuronal excitability. Thus, SP increases the excitability of mDMV neurons by inhibiting K^+^ conductance.

## Introduction

Gastrointestinal toxicity is a common complication of cancer chemotherapy with cytotoxic agents. Nausea and vomiting (NV) are the most frequent and debilitating side effects and are closely related to substance P (SP)-mediated activation of neurokinin-1 receptor (NK1R) in the medulla oblongata (Muñoz and Coveñas, 2014; Yamamoto et al., 2014; Okafor et al., 2017). In experiment with rats, intraperitoneal injection of a cytotoxic agent, cisplatin, elevated the levels of SP in the cerebrospinal fluid for 3 days and induced pica, an indicator of vomiting in rats (Tatsushima et al., 2011). Most neurons that innervate the upper gastrointestinal tract are found in the medial portion of the dorsal motor nuclei of the vagus nerve (mDMV) of the medulla and activate nicotinic acetylcholine receptors onto postganglionic neurons (Berthoud et al., 1991; Hornby, 2001; Travagli et al., 2006). Additionally, high levels of SP-containing axon terminals and NK receptors were identified in the mDMV (Ladic and Buchan, 1998; Le Brun et al., 2008). Therefore, elevated plasma SP levels during chemotherapy are expected to induce NV by activating NK1 receptors on mDMV neurons. However, the effect of SP on the excitability of mDMV neurons is not well understood. While the addition of the most recent NK1R antagonists to standard antiemetic regimen improved control of chemotherapy induced nausea and vomiting (CINV), numerous patients still experienced these effects (Lorusso et al., 2020; review). Further research is needed to understand the mechanisms underlying CINV to treat patients which are resistant to the current antiemetic regimens. Thus, we evaluated the effect of SP on mDMV neurons and investigated the cellular and molecular mechanisms underlying the responses in rat brainstem slices with electrophysiological methods.

## Materials and Methods

### Statement of Ethics Approval

All animal procedures were conducted with the approval of the institutional Animal Care and Use Committee. These procedures were in accordance with the National Veterinary Research and Quarantine Service guidelines of the Republic of Korea. Efforts were made to minimize the number of animals used and their suffering.

### Dorsal Motor Nucleus of the Vagus Slice Preparation

Coronal brain stem slices were taken from 9- to 12-week-old male Sprague–Dawley (SD) rats (KRIB&B, Korea). The rats were deeply anesthetized with isoflurane (5%). The anesthesia level was confirmed by the absence of the flexor withdrawal reflex, after which the chest was compressed to stop the heart. The brainstem was removed and placed in 4°C artificial cerebrospinal fluid (ACSF) composed of (in mM) 125 NaCl, 3 KCl, 1.2 KH_2_PO_4_, 1.2 MgSO_4_, 25 NaHCO_3_, 10 dextrose, and 2 CaCl_2_ and bubbled with 95% O_2_–5% CO_2_. Acidic solutions (pH 6.8) were made by adding HCl. Coronal slices (300 μm thick) containing the DMV were cut using a microtome (VT-1200; Leica, Germany).

### Experimental Design

**FIGURE 1:**
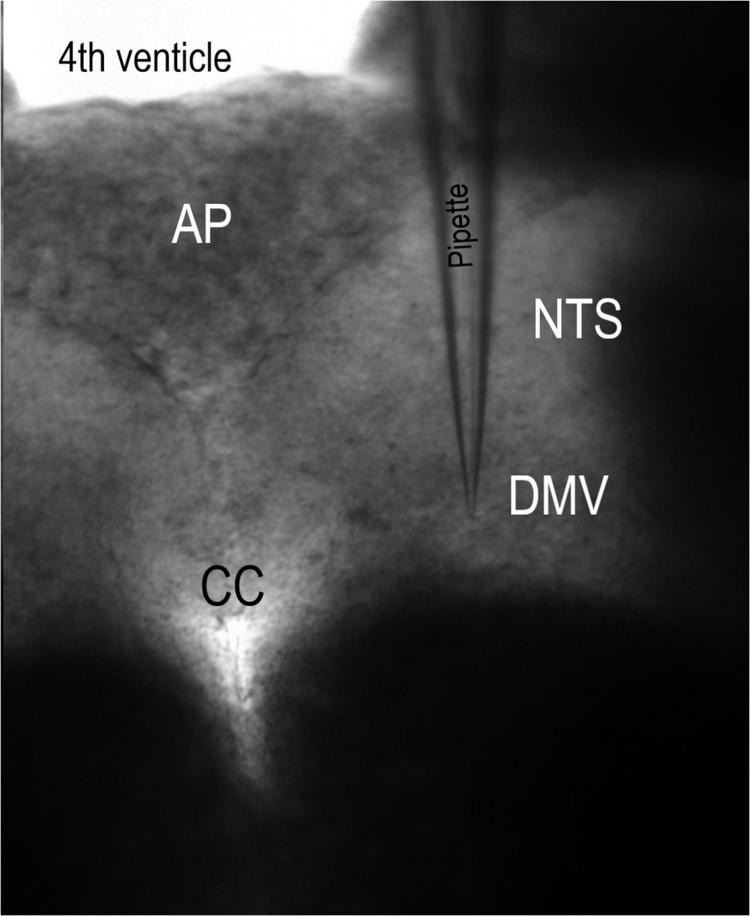
Coronal slice of the brainstem. This 10× magnification micrograph of the rat brainstem slice was retained in a perfusion chamber with polyethylene strands. Pipette, recording electrode. AP, area postrema. CC, central canal. DMV, dorsal motor nucleus of the vagus. NTS, nucleus tractus solitarii.

Slices were secured with a polyethylene mesh in a perfusion chamber and perfused with ACSF at 34–36°C and 307 mOsm. Recordings were then made from DMV neurons. Whole-cell recording pipettes were visually guided to mDMV neurons using infrared illumination and differential interference contrast optics (40X water immersion lens) on a BX51WI microscope (Olympus, Tokyo, Japan) coupled to an infrared-sensitive ZEISS Axiocam 503 mono camera (Figure 1). Whole-cell patch-clamp recordings were carried out using a MultiClamp 700B amplifier (Molecular Devices, Sunnyvale, CA, United States). Voltage-clamp and current-clamp recordings were performed in the conventional whole-cell configuration. Recording electrodes (3–3.5 MΩ) were filled with an intracellular solution containing the following (in mM): 10 NaCl, 40 KCl, 90 K gluconate, 11 EGTA, 1 CaCl_2_, 2 MgCl_2_, 10 HEPES, 2 Na_2_ATP, and 0.2 Na_2_GTP, pH 7.3 (295 mOsm). Under these ionic conditions, Cl^–^ selective currents at the holding potential (-50 mV) will be inward (E_*Cl*_- = -22.4 mV). All recordings were corrected for liquid junction potential. In voltage-clamp mode, 70–75% series resistance compensation was achieved using the series resistance compensation feature of the amplifier. Input resistance measurements were performed by measuring the currents induced by hyperpolarizing steps to -60 and -100 mV. Whole-cell recordings were obtained from 87 visually identified mDMV neurons from different brain slices. Only one DMV slice was made from one animal.

### Materials

All drugs were administered via bath ACSF perfusate. A selective NK-1 receptor antagonist, sendide, was obtained from Enzo (Farmingdale, NY). A selective NK3 receptor antagonist SB223412 (SB); a selective TASK-1 channel blocker ML365, a selective TASK-3 channel blocker, PK-THPP, and 1,2,3,4-tetrahydro-6-nitro-2,3-dioxobenzoquinoxaline-7-sulfonamide (NBQX), 3-([(3,4-dichlorophenyl)methyl]amino]propyl)diethoxymethyl)phosphinic acid (CGP), D-2-amino-5-phosphonovalerate (AP5), SR95531 (gabazine), tetrodotoxin (TTX), and bicuculline were obtained from Tocris Bioscience (Ellisville, MO, United States). SP and picrotoxin were purchased from Sigma–RBI (Natick, MA, United States). Different concentrations of SP were perfused for 6 to 20 min at a flow rate of 1.8–2 ml/min. The synaptic responses over the final 5-min period of each concentration exposure were analyzed.

### Data Analysis

All spontaneous inhibitory and excitatory postsynaptic currents (sIPSCs and sEPSCs) were detected and analyzed from digitized waveforms using MiniAnalysis (Synaptosoft, Decatur, GA), as described previously (Kim et al., 2018). Briefly, except for the determination of frequency rates, events < 4 pA and those with multiple peaks were excluded from waveform analyses and rate calculations. Baseline currents were measured over a 2 ms section of the recorded traces prior to every detected event. For statistical comparisons of sEPSCs or sIPSCs, waveform amplitude, event frequency and baseline values across each group were averaged over the last 5 min of each cumulative concentration step. Cumulative distributions of spontaneous postsynaptic current response amplitudes, frequencies, and baseline currents were compared using Kolmogorov–Smirnov (K–S) non-parametric analysis. For comparing two groups, t test were used. Statistical comparisons of three or more groups were made with one-way ANOVA or repeated-measures ANOVAs (RM-ANOVA) followed by Bonferroni/Dunn *post hoc* testing, when appropriate (Statview 4.57; Abacus Concepts, SAS Campus Drive, Cary, NC). The chi-squared test was used to compare the effect of 0.1 and 1 μM SP on the proportion of firing neurons. All data presented are means ± SDMs. Differences were considered statistically significant at P values of less than 0.05.

## Results

### Substance P Increases γ-Aminobutyric Acid Release and Shifts Holding Current

**FIGURE 2:**
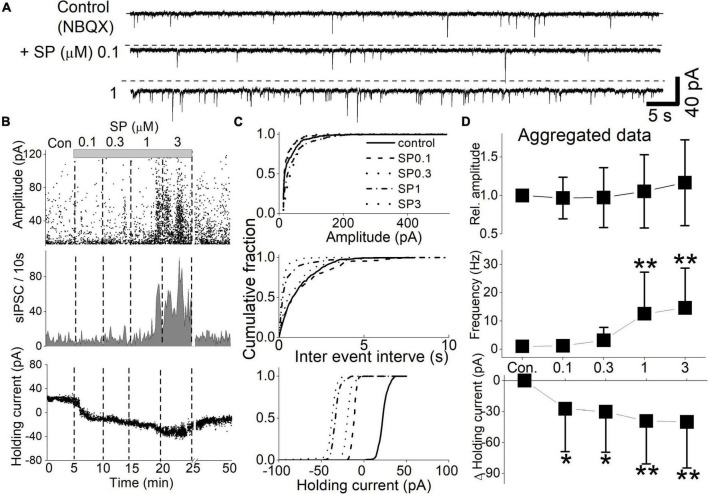
Concentration-dependent SP effect on sIPSCs and holding currents. sIPSCs were isolated by the continuous presence of NBQX (20 μM) at a holding potential of -50 mV. SP (0.1–3 μM) was applied in a cumulating manner. The slices were washed with ACSF for 1 min before higher concentrations of SP ware applied. **(A)** The holding current increased proportionally with the SP concentration. Additionally, the frequency of sIPSCs increased at 1 μM SP. The dashed line indicates the mean holding current before SP treatment. **(B)** Diary plots display the event characteristics of the sIPSCs of representative neurons (upper traces) during the cumulative application of 0.1–3 μM SP. The amplitude (top panel), frequency (middle panel), and holding current (bottom panel) for each detected sIPSC are displayed. **(C)** Corresponding cumulative fractions of event measurements shown for the individual sIPSCs in panel **(B)**. The subpanels show the effect of SP on sIPSC amplitude (top panel), interevent interval (middle panel) and holding current (bottom panel). The baseline holding currents were measured as the absolute current (pA) of the individual responses. **(D)** Summary aggregate data for sIPSC events and concurrent changes in the holding current recorded during application of NBQX (*n* = 9 to 8 neurons). Measurements were normalized by the control amplitude and frequency of the neurons before compiling the averages. Changes in the baseline holding currents were calculated as changes from the control condition in absolute pA.

All recorded neurons had sIPSCs and sEPSCs, as reported earlier (Browning and Travagli, 1999; Lewis and Travagli, 2001). We first examined the effect of SP on GABAergic sIPSCs in the presence of the glutamate receptor antagonist NBQX. Under these conditions, the mean frequency of the sIPSCs was 0.39 ± 0.15 Hz. Application of submicromolar (0.1 and 0.3 μM) concentrations of SP had no effect on sIPSC frequency or amplitude but shifted the holding current more inward (Figures 2A,B). Increasing the SP concentration to micromolar levels (1 and 3 μM) increased the inward level of the holding current and markedly increased the sIPSC frequency. These responses did not fully recover to control levels even after a 25 min washout with ACSF. As a result, brain slices treated with micromolar concentrations of SP were not used further. Even the lowest tested SP concentrations produced a significant inward shift in the holding current (Figure 2C, control vs. 0.1 μM SP, *p* = 0.0001, K-S test). In addition, the sIPSC frequency increased when 1 and 3 μM SP were administrated (Figure 2C, *p* = 0.0001, K-S test). In the 9 tested neurons, SP uniformly shifted the holding current inward, but the sIPSC frequency increased in only 8 of the 9 neurons. Across all neurons, 1 and 3 μM SP significantly increased the sIPSC frequency to 12.6 ± 4.9 and 14.6 ± 4.7 times the control frequency, respectively (Figure 2D, *p* < 0.016, vs. control, RM-ANOVA, *n* = 8). The SP concentrations had no effect on the amplitude of the sIPSC (*P* = 0.9, RM-ANOVA, *n* = 8). At all tested concentrations, the SP-induced holding current shifted inward (*p* < 0.032, vs. control, RM-ANOVA, *n* = 9).

### Substance P Reduces Synaptic Glutamate Release

**FIGURE 3:**
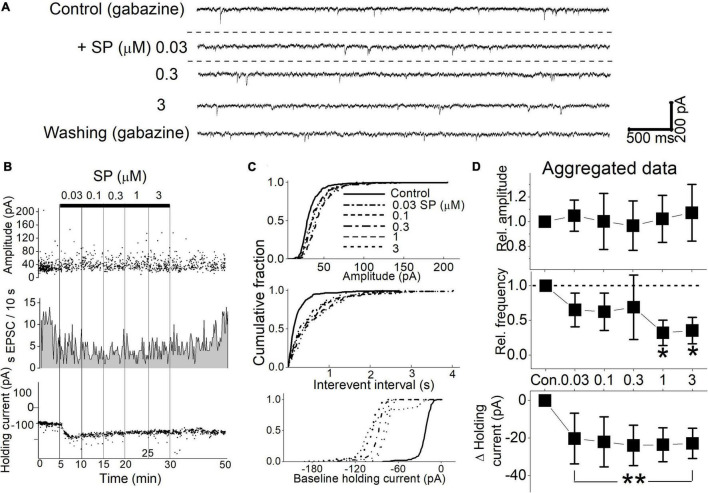
SP effect on sEPSCs and holding currents. **(A)** Glutamatergic sEPSCs were isolated with gabazine (6 μM) and recorded at a holding potential of -50 mV. SP (0.03–3 μM) was applied in a cumulative manner. The slices were washed with ACSF for 1 min before higher concentrations of SP were applied. The dashed line indicates the mean holding current before SP treatment. **(B)** Diary plots display the event characteristics of the sEPSCs of representative neurons (upper traces) during the cumulative application of 0.03–3 μM SP. The amplitude (top panel), frequency value (second panel), and holding current (bottom panel) values for each detected sEPSC are displayed. **(C)** Corresponding cumulative fractions of event measurements shown for each of the individual sEPSCs shown in **(B)**. Each panel shows the effect of SP on the sEPSC amplitude (top panel), interevent interval (middle panel) and holding current (bottom panel). The baseline holding currents were measured as the absolute current (pA) of the individual responses. **(D)** Summary aggregate data for sEPSCs. Measurements were normalized by the control amplitude and frequency of the neurons before compiling the averages. Changes in the baseline holding currents were calculated as changes from the control condition in absolute pA.

The effect of SP on glutamatergic sEPSCs was recorded in the presence of the GABA_*A*_ receptor antagonist gabazine. In the representative neuron shown in Figure 3, the cumulative application of 0.03 to 3 μM SP induced an inward shift in the holding current and reduced the sEPSC frequency (Figures 3A,B). These responses did not fully recover to control levels even after a 20 min washout with ACSF. Statistical analysis revealed that all tested concentrations of SP reduced sEPSC frequency (Figure 3C, *p* < 0.03, K-S test) without affecting the amplitude (*p* > 0.24, K-S test). At the same time, SP induced a significant shift in the holding current at all tested concentrations (*p* < 0.0001, K-S test). Of the 7 tested neurons, SP induced an sEPSC frequency decrease in 6, but shifts in the holding current were observed in only 5 neurons. Summary aggregate data of the sEPSC events show that all tested concentrations of SP reduced the sEPSC frequency below control levels; however, a significant difference was observed only with the application of 1 and 3 μM SP (*p* < 0.03, *n* = 6, RM-ANOVA). SP concentrations had no effect on the amplitude of the sEPSCs (*p* > 0.1, RM-ANOVA, *n* = 7). At concentrations of 1 and 3 μM, SP treatment decreased the sEPSC frequency to 56.1 ± 24.7% and 58.1 ± 23.7% of the control frequency, respectively (Figure 3D, *p* < 0.001, RM-ANOVA, *n* = 6). In 5 of the SP-responsive neurons, all tested concentrations of SP induced an inward holding current (*p* < 0.01, RM-ANOVA, *n* = 5). After 5 min of treatment with 0.03 to 3 μM SP, the baseline holding current shifted inward from the control level at all tested concentrations. In summary, SP suppressed synaptic glutamate release without affect holding current.

### Substance P Induces an Inward Shift of the Holding Current

**FIGURE 4:**
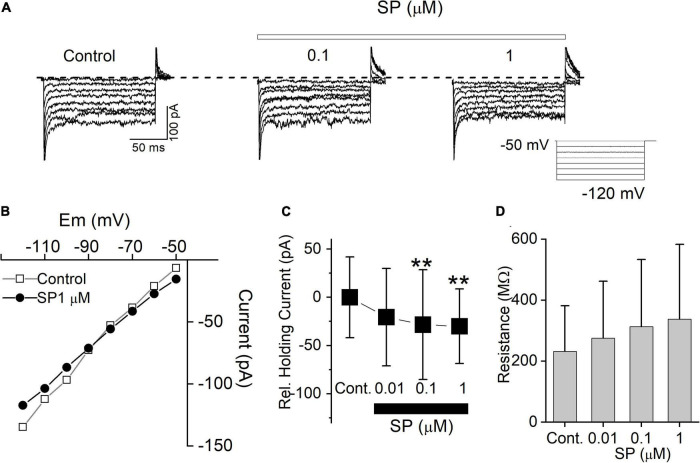
SP inhibits background conductance. **(A)** Current traces from hyperpolarizing voltage steps applied in control conditions and during application of 0.1 to 1 μM SP. Dashed lines indicate control potential. **(B)** The current-voltage (I-V) relation measured from the same cell in **(A)**. **(C)** Average baseline holding current recorded from cells in the control condition and during application of 0.01 to 1 μM SP. **P < 0.01. **(D)** Input resistance in the control and during application of 0.01, 0.1, and 1 μM SP were 231.8 ± 74.9, 275.4 ± 93.1, 313.1 ± 109.9, and 337.2 ± 122.8 MΩ, respectively (*n* = 5).

In this experiment, SP induced an inward shift in the holding current at a resting potential of -50 mV. To examine this more closely, we measured the holding current with increasingly hyperpolarized steps up to -120 mV in the presence of tetrodotoxin (TTX), NBQX and picrotoxin. In all tested neurons (*n* = 5), 0.01 to 1 μM SP reduced the membrane current at all tested holding potentials (Figure 4A). The steady-state current-voltage (I-V) relationships for the control condition and 0.1 μM SP application cross were –89 mV (Figure 4B). The average value of -87.6 ± 0.8 mV (*n* = 5) was close to the potassium equilibrium potential (E_*k*_) -86 mV. Figure 4C summarizes the baseline holding currents for the control conditions and during SP application. SP also increased membrane input resistance in a concentration-dependent manner (Figure 4D). Taken together, the results suggest that the inward shift in the holding current and the increase in input resistance were both driven by the inhibition of K^+^ conductance.

### Acidification Reduces Background Channel Conductance

**FIGURE 5:**
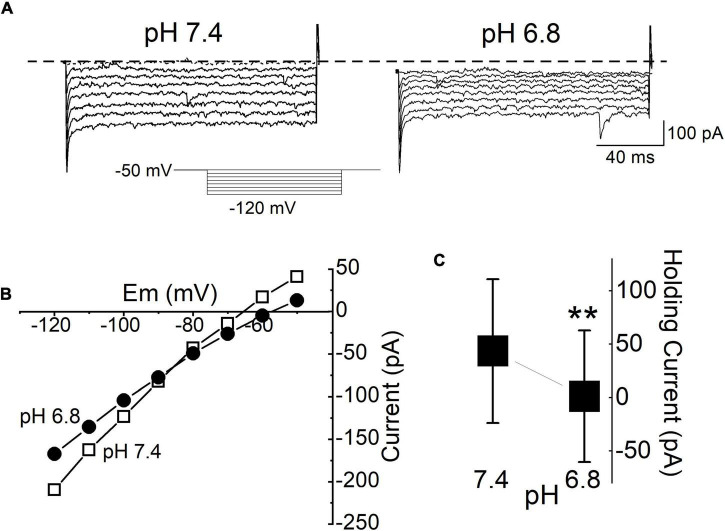
Background K^+^ conductance was reduced by acidification. **(A)** Current traces in response to polarizing voltage steps between -50 and -120 mV from the holding potential of (-50 mV) applied to the same cell during application of pH 7.4 and pH 6.8 ACSF. All traces were recorded during the application of TTX (1 μM). The current response was measured 10 ms after the onset of the voltage step to avoid inclusion of the transient capacitance. **(B)** The I-V relationship measured from the same cell in **(A)**. **(C)** Mean baseline holding current at normal ACSF (pH 7.4) and during acidification (pH 6.8). Each bar and whisker represent the mean ± SDM from 5 different cells. **P < 0.01.

Substance P (SP) reduces the membrane current, but this finding raises the question of the current identity. The tandem pore domain K^+^ channel provides a prominent leak current in many central neurons, and its mRNA is present at high levels in the DMV (Duprat et al., 1997; Talley et al., 2000). Neurotransmitters, including SP, inhibit the tandem pore domain in a weak inwardly rectifying (TWIK)-related acid-sensitive potassium (TASK) channel, similar to the effect of acidification (Talley et al., 2000). Thus, we tested the pH sensitivity of the background-leak current in our mDMV neurons by measuring the steady state I-V relationships in the continuous presence of TTX, gabazine (GZ), and NBQX. Acidification of the extracellular solution (pH 6.8) from the control condition (pH 7.4) reduced the current responses at all voltage steps, in 78% of the tested neurons (*n* = 14/18, respectively; Figure 5A). The mean holding current at pH 6.8 was significantly less than that of the control condition at pH 7.4 (Figure 5C). The mean holding currents at pH 7.4 and 6.8 were 43.5 ± 67.3 and 1.1 ± 61.6 pA, respectively. The I-V relationship had a reversal potential of -86 ± 0.5 mV (Figure 5B).

### The TWIK-Related Acid-Sensitive K^+^ Channel Affects Holding Current

**FIGURE 6:**
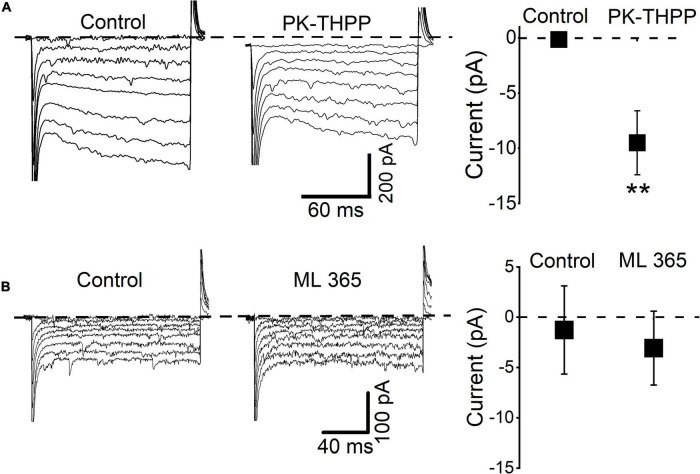
DMV neurons express a leak K^+^ conductance similar to TASK-3 neurons. **(A)** Current traces from hyperpolarizing voltage steps (increment: -10 mV, range: from -50 to -120 mV) applied in control conditions and during application of PK-THPP (0.1 μM). Average baseline holding current recorded from cells in the control condition and during application of PK-THPP **P < 0.01 (*n* = 5). **(B)** Current traces from hyperpolarizing voltage steps applied in control conditions and during application of ML365 (10 nM). Dashed lines indicate current level at -50 mV.

The above results suggest that TASK channels participate in the SP-induced inward shift in the holding current and reduced input resistance. Indeed, high mRNA levels of TASK-1 and TASK-3 are found in the rodent DMV, while other two-pore domain K^+^ channel transcripts are expressed at lower levels (Talley et al., 2001). Hence, we characterized the background K*^+^* current using selective inhibitors of TASK-1 and TASK-3. Application of a selective TASK-3 blocker, PK-THPP, induced similar responses as acidification (Figure 6). In all tested neurons (*n* = 5), the application of PK-THPP shifted the baseline holding current inward by approximately 9.6 ± 2.9 pA and reduced the membrane current. Meanwhile, application of a TASK-1 inhibitor, ML365, did not significantly affect the baseline holding current or membrane resistance (*n* = 5). Taken together, the results show that TASK-3 channels are likely involved in the regulation of excitability of mDMV neurons.

### Substance P Acts on Different NK Receptors

**FIGURE 7:**
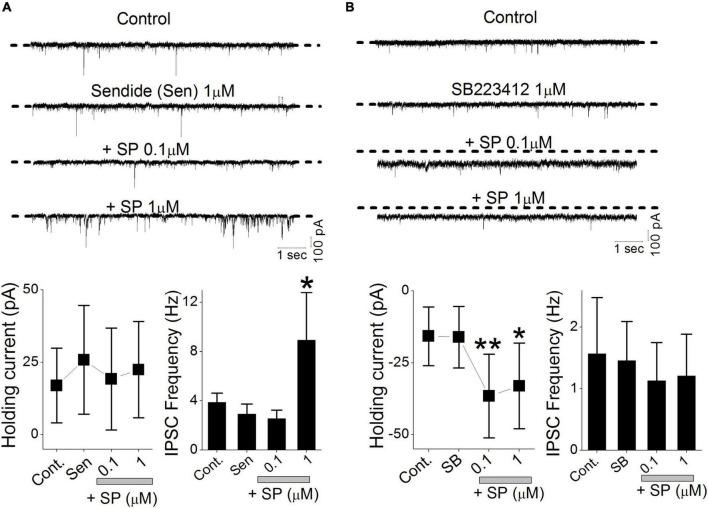
NK receptors affect SP-mediated presynaptic GABA release and postsynaptic holding current. sIPSCs were isolated by recording in the continuous presence of NBQX (20 μM). **(A)** Original traces show the effect of sendide alone, sendide + 0.1 μM SP, and sendide + 1 μM SP. All traces were obtained from a single neuron. The histograms summarize the holding currents and mean frequencies of the sIPSCs from 6 different neurons. **(B)** Original traces show the effects of 1 μM SB223412 (SB), SB + 0.1 μM SP, and SB + 1 μM SP. All traces were obtained from a single neuron. The histograms summarize the holding currents and mean frequencies of the sIPSCs from 6 different neurons. Each point and error bar represent the mean ± SDM from 9 different cells. **P < 0.01, * *p* < 0.05.

Substance P (SP) binds to all three NK receptor subtypes (NK1, NK2 and NK3), but NK1 has the highest affinity for SP. The expression of NK1 and NK3 receptors has been reported in rat DMV but not that of NK2 (Saffroy et al., 2003). Our lowest SP concentration (0.03 μM) preferentially induced a holding current shift without altering the sIPSC frequency. To isolate SP action, we tested the NK1 selective antagonist sendide, which affects SP-mediated responses (Lewis and Travagli, 2001). Sendide (1 μM) alone did not affect the holding current or frequency of the sIPSCs (Figure 7A). In the continuous presence of sendide, neither 0.1 nor 1 μM SP affected the holding current (RM-ANOVA, *p* > 0.45, *n* = 8). In contrast, 1 μM SP significantly increased the sIPSC frequency despite the presence of the NK1 antagonist in 6 of 8 tested neurons (RM-ANOVA, *p* = 0.03). The mean frequency of the sIPSCs measured before and after application of 1 μM SP was 2.9 ± 0.8 Hz and 8.9 ± 3.86 Hz, respectively (*n* = 6). In separate experiments, the effect of NK3R on the SP-mediated holding current and sIPSC frequency was tested in 6 different neurons. The NK3R antagonist, SB223412 (1 μM, SB), alone did not affect the holding current or frequency of the sIPSCs (Figure 7B). In the presence of SB (1 μM), perfusion with 0.1 and 1 μM SP shifted the holding current inward (RM-ANOVA, ** *p* < 0.003, * *p* < 0.012, *n* = 6). Neither 0.1 nor 1 μM SP induced any significant changes in sIPSC frequency (*p* > 0.4, *n* = 6).

### Heterosynaptic Regulation of Glutamate Release

**FIGURE 8:**
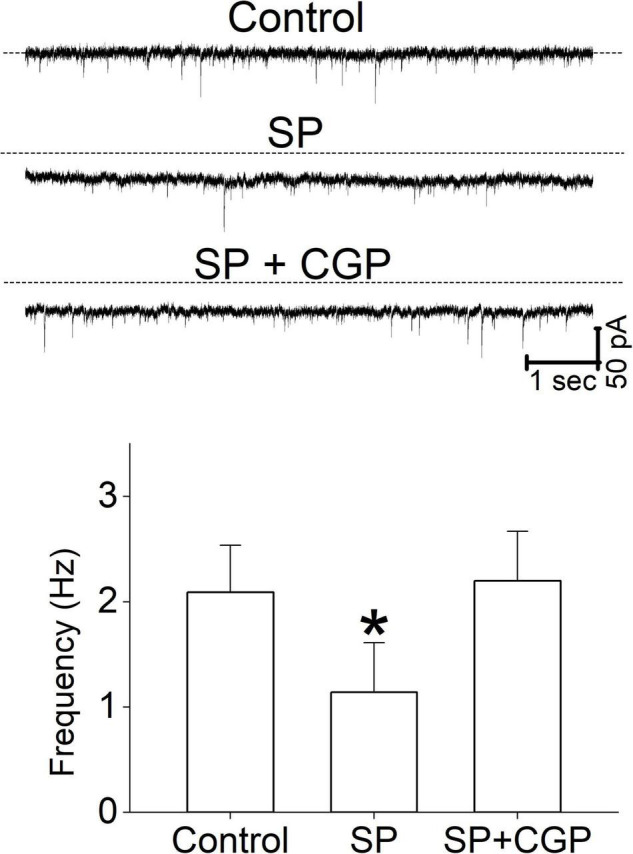
The effect of a GABA_*B*_ receptor antagonist on sEPSC frequency. Representative current traces from a single neuron show the effect of SP (1 μM) and CGP (5 μM) on sEPSCs. The sEPSC frequency data were obtained from 5 different neurons. All columns and error bars indicate the mean ± SDM, * *P* < 0.05.

In this study, micromolar concentrations of SP facilitated synaptic GABA release by activating NK3R and suppressed synaptic glutamate release via an unknown mechanism. In the neuronal circuitry of the brain stem, glutamatergic and GABAergic synaptic responses are often influenced by metabotropic receptor-mediated heterosynaptic modulation (Jin et al., 2004; Fernandes et al., 2011). To test whether a GABA_*B*_ receptor antagonist altered glutamate release, we tested the effect of SP in the presence of the selective GABA_*B*_ receptor antagonist CGP. The application of SP under control conditions decreased sEPSC rates, and the addition of the CGP blocked the SP-induced reduction in sEPSCs (Figure 8). The mean frequency of the sIPSC at control, SP, and SP + CGP was 2.1 ± 0.45, 1.14 ± 0.47 and 2.2 ± 0.47, respectively. Thus, our results imply that the SP-mediated increase in the release of GABA reduced synaptic glutamate release from glutamatergic neurons via GABA_*B*_ receptor activation. Perfusion of 1 μM SP reduced the sEPSC frequency to 60% of the control frequency (Figure 8, *p* = 0.045, RM -ANOVA, *n* = 5). In the same set of neurons, however, SP failed to reduce the sEPSC frequency in the presence of 5 μM CGP (*p* = 0.78, RM -ANOVA, *n* = 5). However, in the same experiments SP application shifted the holding current inward. The mean holding current at control, SP, and SP + CGP was 10.4 ± 15.8 pA, 31.6 ± 23.8 pA and 30.8 ± 21.3 pA, respectively.

### Substance P Increases Neuronal Excitability

**FIGURE 9:**
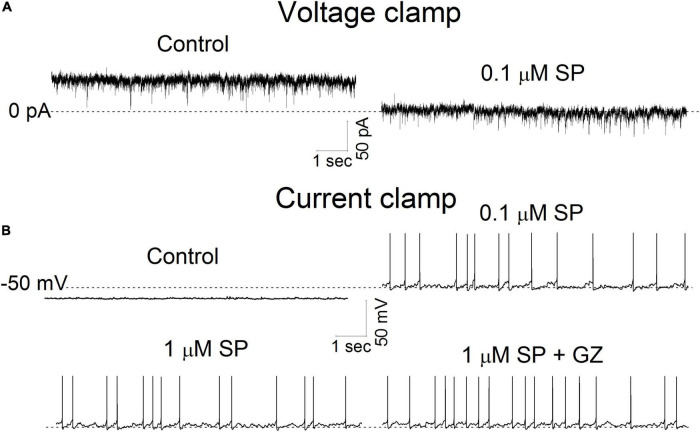
The effect of SP on holding current and membrane potential. **(A)** Current traces were recorded in voltage-clamp mode before and during application of 0.1 μM SP at a holding potential of -50 mV. The dotted line represents 0 pA. **(B)**. Membrane potential traces were recorded before and during a 5 min application of SP at 0.1, 1 μM and 1 μM + gabazine (GZ) 6 μM. All traces were obtained from the same neuron. The dotted line represents -50 mV.

**FIGURE 10:**
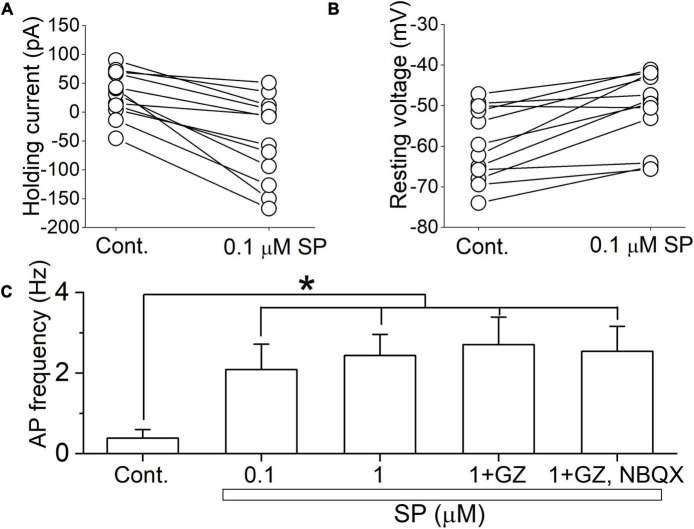
SP-induced depolarization is a major contributor to action potential firing. **(A)** The holding current was recorded before and during a 5 min application of 0.1 μM SP at a holding potential of -50 mV (*n* = 12). **(B)** The resting membrane potential was recorded from the same set of neurons as in **(A)** in current clamp mode without current injection. Connected points represent measurements from individual cells before and after application of 0.1 μM SP. **(C)** The action potential frequency was calculated under the control conditions and after application of 0.1 and 1 μM SP, 1 μM SP + gabazine, and 1 μM SP + gabazine + NBQX (20 μM) (*n* = 12). All columns and error bars indicate the mean ± SDM, * *P* < 0.05 vs. control.

These multiple effects on synaptic transmission as well as membrane conductance indicate that SP may alter neuronal excitability. To assess this in single neurons, we exposed neurons first to 0.1 μM SP under voltage-clamp and then followed that with current-clamp recordings of the responses. Application of 0.1 μM SP induced an inward current under voltage-clamp conditions (Figure 9A). During the current-clamp, 0.1μM SP both depolarized and elicited action potentials, as shown in the representative neuronal tracings in Figure 9. A high concentration of SP (1 μM) failed to increase the firing rate (Figure 9B). In twelve tested neurons, application of 0.1 μM SP shifted the holding current inward (Figure 10A). The mean holding current before and during 0.1 μM SP application was 32.3 ± 11.5 pA and -47.9 ± 21.3 pA, respectively. During the current-clamp, the neurons averaged a resting potential of -59.7 ± 2.6 mV in the control condition and 25% (*n* = 3/12) spontaneously fired action potentials. Application of 0.1 μM SP depolarized the mean resting membrane potential to -50.3 ± 2.6 mV; 75% of neurons fired action potentials in this condition (Figure 10B, *n* = 9/12). The application of 1 μM SP did not further affect the firing rate (*p* > 0.6, n = 12, RM ANOVA) or the percentage of firing neurons (*p* = 0.4, chi-square test). In the presence of 1 μM SP, the addition of GZ (*p* = 0.38, *n* = 11, paired *t* test) or the coapplication of GZ and NBQX (*p* = 0.65, *n* = 10, paired *t* test) failed to induce significant changes in action potential frequency (Figure 10C).

## Discussion

In this study, we tested the response of mDMV neurons to SP to elucidate the mechanism of action underlying CINV at the molecular and cellular levels. Submicromolar concentrations of SP induced an inward shift in the baseline current by decreasing membrane K^+^ conductance. SP-induced responses were mimicked by acidification of the extracellular solution and application of TASK-3 blockers but were blocked by application of NK1R antagonists. Micromolar concentrations of SP increased synaptic GABA release in an NK-3R activity-dependent manner but reduced the frequency of spontaneous EPSCs in a GABA_*B*_ receptor activity dependent manner. In a current-clamp experiment, application of submicromolar SP caused membrane depolarization and firing activity in mDMV neurons. However, no significant changes were observed upon further elevation of the SP concentrations to micromolar ranges.

NK1 receptor antagonists have been used in combination with corticosteroids and 5-HT3 receptor antagonists to prevent CINV associated with highly emetogenic agents. However, a significant number of cancer patients still suffer from CINV (Dranitsaris et al., 2017; Nurgali et al., 2018). Hence, the development of new classes of antiemetic drugs is needed. In our experiment, SP action on NK1R receptor increased the excitability of mDMV neurons by reducing K^+^ efflux through TASK-3 channels. Therefore, the activity of TASK-3 plays a decisive role in the regulation of the excitability of mDMV neurons; a substance that activates TASK-3 is expected to suppress CINV by hyperpolarizing mDMV neurons. Interestingly, TASK-3 channel activators are already in clinical use for general anesthesia, rather than antiemetic purposes. For example, some halogenated volatile anesthetics (e.g., halothane and isoflurane) are known to induce anesthesia by hyperpolarizing membrane potential via activation of TASK channels (Meuth et al., 2009; Muhammad et al., 2010; Yao et al., 2017). However, systemic use of these anesthetics is known to cause NV rather than inhibit it. Therefore, it appears that targeted delivery of TASK-3 activators to mDMV is required to inhibit CINV. However, this is currently impossible.

A more feasible way to inhibit CINV is to increase the K^+^ conductance of TASK-3 channels by alkalizing the blood. The open probability of the TASK-3 channel increases with alkalization (Rajan et al., 2000). Thus, elevation of the blood plasma pH is expected to interrupt SP-induced activation of mDMV neurons. Typically, pH levels in the blood are determined by the partial pressure of oxygen (PO_2_) and carbon dioxide (PCO_2_). A Japanese research group showed that mild hyperthermia induced alkalization of blood pH in advanced cancer patients by increased PO_2_ and decreased PCO_2_ in the blood (Ohishi et al., 2009). Their findings showed that 30 min of hyperthermia (39.5°C) increased blood pH from less than 7.4 to pH 7.7; this improved clinical responses but effects on gastrointestinal function were not evaluated. In addition, electrophysiological recording of the TASK-3 channel showed that the open probability of the K^+^ channel continuously increases during elevation of the pH of from 6 to 8 (Kim et al., 2000). Taken together, our findings suggest the possibility of inhibiting CINV resistance to antiemetic regimens by increasing body temperature. However, further clinical studies are needed to elucidate the potential of thermotherapy-induced antiemetic action.

In this experiment, SP-mediated tonic current shifts and spontaneous increases in GABA release were inhibited by NK1 and NK3 receptor antagonists, respectively. These results imply segregated expression of NK receptors on different neurons. An immunohistochemistry study of DMV neurons showed that most NK1-positive neurons were cholinergic, whereas NK3-positive neurons were non-cholinergic (Le Brun et al., 2008). In addition, NK1R expression was identified in retrogradely labeled vagal efferent DMV neurons that innervate the stomach or duodenum (Ladic and Buchan, 1996; Krowicki and Hornby, 2000; Lewis and Travagli, 2001). Furthermore most efferent cholinergic DMV neurons exclusively innervate the stomach (Altschuler et al., 1991; Berthoud et al., 1991; Ladic and Buchan, 1996). In contrast, the NK3 receptor is predominantly expressed in GABAergic inhibitory interneurons (Le Brun et al., 2008).

## Data Availability Statement

The original contributions presented in the study are included in the article/supplementary material, further inquiries can be directed to the corresponding authors.

## Ethics Statement

The animal study was reviewed and approved by Institutional Animal Care and Use Committee of the Kyung Hee university.

## Author Contributions

EY and WK performed the experiments and drafted the manuscript. YP and Y-HJ generated the concept of the study and supervised the work. Y-HJ contributed to drafting and revising the manuscript. All authors of this manuscript have read and approved the manuscript for submission.

## Conflict of Interest

The authors declare that the research was conducted in the absence of any commercial or financial relationships that could be construed as a potential conflict of interest.

## Publisher’s Note

All claims expressed in this article are solely those of the authors and do not necessarily represent those of their affiliated organizations, or those of the publisher, the editors and the reviewers. Any product that may be evaluated in this article, or claim that may be made by its manufacturer, is not guaranteed or endorsed by the publisher.
